# How structural and symbolic violence during resettlement impacts the social and mental wellbeing of forced migrant women: the lived experiences of Arabic speaking survivors of IPV resettled in Melbourne, Australia

**DOI:** 10.1186/s13031-022-00494-6

**Published:** 2022-11-11

**Authors:** Jeanine Hourani, Yara Jarallah, Karen Block, Linda Murray, Jasmin Chen, Maria Hach, Cathy Vaughan

**Affiliations:** 1 Melbourne School of Population and Global Health, The University of Melbourne, Melbourne, Australia; 2 Te Ngira Institute for Population Research, University of Waikato, Hamilton, New Zealand; 3 College of Health Sciences, Massey University, Palmerston North, New Zealand; 4 Multicultural Centre for Women’s Health, Melbourne, Australia

**Keywords:** Structural violence, Symbolic violence, Intimate partner violence, Mental health, Forced migrant

## Abstract

Forced migrant women experience high levels of violence across their journeys and violence can be characterised as having three overarching forms: structural, symbolic, and interpersonal. It is important to understand the intersecting nature of gendered forms of symbolic, structural and interpersonal violence, and their impact on the mental health of forced migrant women in order to develop holistic IPV and resettlement programs and interventions. This article adopts an ecological framework of violence and qualitative methods with mental health service providers and survivors of IPV to understand the intersections of different forms of violence and their impact on mental health as they relate to the lived experiences of Arabic-speaking forced migrant survivors currently residing in Melbourne, Australia. Our research has three key findings: (1) that forced migrant women living in Melbourne, Australia experience intersecting forms of violence during resettlement (2) Structural and symbolic violence against forced migrant women persists regardless of marital status (3) Autonomy and independence plays a vital role in the mental health and wellbeing of forced migrant women. Our findings reveal that structural and symbolic violence increase the risk of IPV for forced migrant women during resettlement and that even when forced migrant women leave IPV situations, structural and symbolic violence persist and exacerbate mental ill-health. This article also reveals the importance autonomy and independence in both the perpetration of violence and in healing and recovery.

**Supplementary Information:**

The online version contains supplementary material available at 10.1186/s13031-022-00494-6.

## Background

Over the past decade, forced migration has doubled and become increasingly feminised [28]. While forced migrant experiences are diverse and complex, it has been well documented by organisations like Doctors of the World and the Women’s Refugee Commission that forced migrant women experience high levels of violence across their journeys [14, 26, 36].

The Australian Humanitarian Settlement Program (HSP) settles approximately 14,000 refugees per annum, including through Women at Risk visas. In 2015, the Australian Government settled an additional 12,000 Syrian refugees and prioritised women, children and families. In addition to women arriving through the HSP, there are many other routes through which forced migrant women may arrive in Australia; this includes asylum seeking women, women arriving on spousal visas, and women arriving through other channels but were fleeing violence or persecution, nonetheless. To account for this variation, this paper adopts a broad definition of ‘forced migrant women’ to encompass all women fleeing violence or persecution. For example, some of the survivors interviewed in this paper had migrated from countries with ongoing violence but had arrived in Australia through spousal visas.

Violence can be characterised as having three overarching forms that are intersecting yet distinct: structural, symbolic and interpersonal [14, 23, 37]. These forms of violence are dynamically linked across space and time through fluid social, political and economic processes that shape violence in both private and public spheres by different actors [19].

Structural forms of violence are built into the fabric of society and upheld by institutions to create and maintain inequalities within and between social groups on the basis of gender, sexuality, ethnicity, religion, ability, socioeconomic status, migration and visa status, and other characteristics [24]. Structural violence results in differential access to information, resources, voice, agency and representation [17]. Forced migrants are subjected to structural violence such as restrictive immigration policies, lack of social support, inadequate opportunities to learn the host-country language, lack of employment opportunities, lack of financial support, all of which place forced migrants at an increased risk of poverty and destitution [11, 16, 21, 22, 24, 26, 30, 32, 38]. In Australia, the magnitude of structural violence experienced by forced migrants differs based on visa status. For example, arrivals through the HSP are legally entitled to health and welfare services while people seeking asylum have limited entitlements. Regardless of visa status, structural barriers to accessing services (such as availability of interpreters) exist for migrants in Australia.

The term symbolic violence was coined by Pierre Bourdieu [3] and is used to describe the ideologies, behaviours, words, and non-verbal communications that produce, enable and reproduce power relations [3, 24, 31]. Structural violence against forced migrants is underpinned, enabled, and legitimised by xenophobia, Islamophobia, and anti-immigrant ideologies during resettlement [2, 5, 6, 14, 22]. Racism in Australia is underpinned by the White Supremacist settler colonial history of the Nation. Since colonisation, different waves of migrants have been subjected to varying levels of racism and xenophobia. The year 2001 marked an increase in the politicisation and vilification of forced migrants in Australian domestic politics that saw an increase in racist, xenophobic and islamophobic national policies and local attitudes. Although Melbourne is generally considered the most progressive major city in Australia, racism and anti-immigrant sentiment remains pervasive.

Interpersonal violence encompasses violence perpetrated by and between individuals, including between family members and intimate partners [37]. There has been found to be an increased risk of intimate partner violence (IPV) for forced migrant women and these have structural and symbolic root causes [1, 7, 8, 29, 35]. For example, an Australian study found that forms of structural violence such as lack of employment opportunities or lack of financial support can challenge masculinities within families or households by making men feel as though their role as ‘breadwinner’ has been undermined [38]. Such structural challenges of symbolic patriarchal norms has been found to increase the risk of IPV for forced migrant women [16, 32, 34, 35].

Structural and symbolic violence during resettlement have been found to contribute to poorer mental health for forced migrants with forced migrant women being at significantly greater risk of mental ill-health than men [14, 16, 29, 30]. At the same time, the relationship between mental health and IPV is complex and bidirectional; women with mental illness experience higher rates of IPV and women who have experienced IPV have poorer mental health outcomes [10, 12].

It is important to understand the intersecting nature of gendered forms of symbolic, structural, and interpersonal violence, and their impact on the mental health of forced migrant women in order to develop holistic IPV and resettlement programs and interventions that prevent and respond to all forms of violence against forced women [13, 25, 27]. This article subsequently adopts an ecological framework of violence [24] which is rooted in the understanding that violence occurs in the context of the larger socially, culturally, politically, economically, and historically constructed systems. Specifically, this article understands violence against forced migrant women to operate at the intersection of constructions of patriarchy and race. Structurally, patriarchy organises social institutions and relationships within a hierarchy that enables men to maintain positions of power. Similarly, constructions of race organise social institutions and relationships within a hierarchy that enables White domination and power. Symbolically, constructions of patriarchy and race validate and legitimise such subordination of women and racialised minorities respectively. Our research locates such constructions of patriarchy and race within an intersectional feminist framework [9] which understands patriarchy and race to exacerbate disadvantage, thus compounding the structural and symbolic oppression of forced migrant women [9, 20]. In line with this framework, we acknowledge that patriarchy and race must not only be considered together when understanding violence against forced migrant women, but they must also be addressed and overcome together. This article thus adopts qualitative methods to understand the intersections of different forms of violence and their impact on mental health as they relate to the lived experiences of Arabic-speaking forced migrant survivors of IPV currently residing in Melbourne, Australia.

## Methods

### Data collection

The data collection methods employed in this study were two participatory workshops with forced-migrant survivors, six in-depth interviews with forced migrant survivors, and four in-depth interviews with mental health service providers.

The mental health service providers were recruited from a mental health service provider in Melbourne who specialise in providing counselling and mental health support to refugees and people seeking asylum. All four interviewees were females and their official roles within the organisation were ‘Counsellor Advocate’. The in-depth interviews with service providers were conducted in English by Author 2, were approximately 60-min in length and were audio-recorded following informed consent. The service provider interview guide can be found in Additional file 1.

The forced migrant survivors for the participatory workshop were recruited through a resettlement service organisation based in Melbourne. The partner organisation promoted the workshop to their clients that were known survivors of IPV. Those survivors who were interested in taking part then signed up to and attended either one of the participatory workshops. The workshops were conducted in Arabic and were co-facilitated by Author 1 and Author 2, both of whom are native Arabic speakers.

There were 14 participants in each workshop, making up a total of 28 survivors. In line with community-based participatory research principles, the workshops aimed to centre individual and community strengths, enable co-learning, and balance research and action [15]. More specifically, the purpose of the participatory workshops was to understand the context of both settlement and mental health for forced migrant survivors of IPV living in Melbourne. These workshops called on participants to use their own words, expressions, and idioms for talking about resettlement, mental health, and wellbeing. During the workshop, participants were divided randomly into groups of 4–5 and were guided through 3 different activities that explored their understandings of life in Australia, mental health, and healthy families. English translations of these activities can be found in Additional file 2.

The worksheets were collected at the end of each workshop and were thematically analysed as artefacts alongside audio tapes of the workshop that were recorded following informed consent. The analysis of workshop artefacts and audio-recordings was used to create a shared vocabulary between the researchers and the survivors when talking about experiences of resettlement and mental health. This shared vocabulary was then used to develop the interview guides and informed analysis of the data. Enabling research participants to determine the vocabulary used throughout this research project enabled a better understanding and exploration of women’s lived experiences of forced migration and mental health.

During the workshops, participants were informed that in-depth interviews were also being conducted and that they could opt in by providing their consent. Research participants who opted in were subsequently contacted by telephone to arrange a time and place for the interview. The in-depth interviews with survivors were conducted in Arabic by Author 1 and Author 2, were approximately 90-min in length and were audio-recorded following informed consent. The survivor interview guide can be found in Additional file 3.

The qualitative methods used for this study are a subset of a broader project that explored the role of multicultural and settlement services in supporting women experiencing violence in Australia between early 2018 to mid-2019. The research protocol was approved by the University of Melbourne Human Research Ethics Committee (ethics ID 1852384). The full protocol employed for this project and the project findings have been published by Vaughan et al. [33].

### Data analysis

Data was analysed using Braun and Clarke’s well-established approach of thematic analysis which involves identifying, analysing, and reporting patterns within data [4]. The artefacts collected during the participatory workshops were consolidated so that trends and patterns could be identified in the data. The initial themes identified from the artefacts were then used to further explore over-arching themes. For example, when completing the ‘Life in Australia’ worksheet, many groups wrote about trouble securing employment and this was further explored through the in-depth interviews.

Upon completion of all the in-depth interviews, audio recordings were transcribed and, where relevant, translated to English. Due to the sensitive nature of the data and the importance of confidentiality, translation and transcription was completed by Author 1 who was present during all interviews and thus also could reflect on non-verbal cues by cross-referencing field notes during the translation process. This is underpinned by the understanding that translation is not merely verbatim replacement of an Arabic word with an English one; rather, this approach acknowledges the importance of considering idioms, non-verbal cues, and the specific cultural context when translating data.

Specific codes (or sub-themes) were developed from the interview transcripts and these were then clustered into larger themes. A coding framework was created that included organising themes, basic themes, and sub-themes. The coding framework is provided in Additional file 4. Data was coded using the coding framework with the support of NVIVO software.

## Results

This section begins by providing an overview of survivor characteristics to situate the research. We then go into an overview of each of the three themes that underpin the research findings:

1. Intersecting forms of violence experienced by forced migrant women during resettlement
2. The persistence of structural and symbolic violence against forced migrant women, regardless of their marital status
3. The role that autonomy and independence play in the mental health and wellbeing of forced migrant women

### Survivor characteristics

There were 14 survivors that attended each workshop, with a total of 28 women across both workshops. Survivors’ age ranged from 32 to 56 years old, with the mean age being 40 years old. Workshop participants had been living in Australia between 6 months and 5 years, with the median length of time in Australia being two years. The majority of the women (80%) were married, and the large majority (88%) had attained at least a Middle School Education, with 40% having attained a University education. The survivors interviewed were from Syria, Iraq, Lebanon, and Egypt, and were in their 30 s and 40 s. All six women had experienced some form of IPV. Of the six women, one was divorced, three were separated from their husbands, while two were still cohabiting with their husbands. All the women interviewed had children. Half the women had run their own businesses in their country of origin. Half the women described experiences of forced marriage, with one of these women also disclosing early marriage. An overview of the characteristics of survivors who took part in the workshops and in-depth interviews is shown in Table 1.

**Table 1:** Characteristics of survivors

Characteristics	Workshop participants (*N* = 28)	Interview participants (*N* = 6)
*Age*		
31–35	8	0
36–40	8	1
41–45	4	2
46–50	6	0
51–55	1	0
56–60	1	2
No response	0	1
*Country of origin*		
Iraq	9	2
Syria	15	1
Lebanon	1	2
Egypt	1	1
No response	2	0
*Relationship status*		
Married	23	2
Separated	3	3
Divorced	2	1
*Length of time in Australia*		
< 1 year	2	0
1—2 years	6	1
3—4 years	11	0
> 4 years	7	4
No response	2	0
*Educational attainment*		
Primary School	3	1
Middle School	8	2
High School	7	1
University degree	10	1
No response	0	1

### Intersecting forms of violence

Many women mentioned incidents of intersecting forms of violence. For example, in the quote below, an interview participant articulates how symbolic violence (racism) means that the visa status of herself and her husband prevented them from being able to work (structural violence). This meant that her husband lost his traditional ‘breadwinner’ role which challenged his notion of masculinity. In response, her husband attempted to exert control inside the house through perpetrating IPV. This example thus shows us how symbolic and structural violence intersect to increase the risk of IPV against forced migrant women.

“My husband used to get frustrated about not being able to provide for his family. When he got frustrated about that, he used to hit me”

– Survivor from Syria

In another example, an interviewee describes her experience of facing racism in public. In this example, the survivor felt that she could not respond to symbolic violence (racism) due to her precarious visa status and pending visa application (a form of structural violence); structural violence thus silenced the survivor from feeling confident enough to stand up for herself against racism.

“We were on the tram and my husband and I were speaking Arabic but, of course, I can understand a little bit of English so I could understand people on the tram… what they say about us. Once, there was a woman saying things like ‘why have they come to our country?’ and that ‘they take all of our tax money and jobs’. She thought I didn’t understand what she was saying so I turned around and said ‘you should be careful about what you say because you never know who’s going to understand you’. At first, I never used to say anything when people were rude. I just used to cry.... When I didn’t have permanent residency, I felt weak and powerless”

– Survivor from Egypt

Similarly, a workshop participant articulated how patriarchal understandings of masculinity and femininity (underpinned by symbolic norms) can increase the risk of IPV against forced migrant women despite experiences of structural violence (in this case unemployment) being shared amongst forced migrant men and women.

“A lot of challenges faced by men and women are the same but present differently. For example, if a man isn’t working, he ends up sitting at home and fighting with his wife and children, and then women are expected to decompress the pressure in the house”

– Workshop Participant

This quote above thus illustrates that although not all structural violence is gendered, it can become gendered when it intersects with symbolic forms of violence—in this case patriarchy.

In some cases, women described situations in which forms of structural and symbolic factors provided perpetrators with more avenues to perpetrate IPV. For example, some women described perpetrators finding out what services are available to forced migrants and not sharing this information with their partners. This was always enabled by structural factors that created and maintained power imbalances between the perpetrator and survivor. For example, some women were on refugee visas while their husbands were citizens or permanent residents—this often meant that perpetrators had greater access to social services than forced migrant women which created unequal access to resources. In other examples, the women had little grasp of the English language while the husband was fluent which created unequal access to information. Perpetrators were thus able to take advantage of this power dynamic to prevent women from seeking support services, thus further exacerbating structural violence. In the example below, the interview participant describes having information withheld from her from her husband who is an Australian citizen born and raised in Australia.

“He never used to tell me anything. He’s sneaky. He used to use all the services we have available to us and not tell me about them so that I couldn’t access them”

– Survivor from Lebanon

The evidence provided in this section illustrate how forms of structural violence perpetrated by the state (such as precarious visa status and unemployment) which is not necessarily gendered, intersects with symbolic violence (such as racism and patriarchy) which can enable IPV against forced migrant women to be perpetrated. In some cases, the violence perpetrated against them is also structural in nature (such as preventing women from being able to access services) which creates a cycle of violence and abuse against forced migrant women.

### Structural and symbolic violence persist

The evidence presented in the previous section illustrates that structural and symbolic violence can increase the risks of IPV being perpetrated against forced migrant women. However, forced migrant women spoke about their experiences of structural and symbolic violence even after they had left abusive relationships. This illustrates that while there is a reinforcing relationship between structural and symbolic forms of violence and IPV, structural and symbolic forms of violence against forced migrant women persist regardless and continue to negatively impact the mental health of forced migrant women. In the quote below, a survivor describes how the source of her mental ill-health shifted from IPV to structural violence upon leaving her perpetrator.

“What affects my mental health the most now that I’ve left him, is that I don’t work and what would happen if I was to be cut off from Centrelink. Before, I had poor wellbeing because of him. Now, I have poor wellbeing because of financial stress. Not having a job has really impacted my mental health. I have become in a state of depression because of it. There are days where I can’t leave the house because I feel like I’m suffocating”

– Survivor from Lebanon

Another woman described the stigma (symbolic violence) she faced from her community after she left her perpetrator and the trouble she had making friends due to this.

“No one from our community really wants to befriend a divorced woman. My situation here makes it very hard. I thought here people would be more open minded but that’s not the case”

– Survivor from Iraq

Many women who are still married to and living with their perpetrators also focused on structural violence during their interview. For example, the survivor quoted below consistently described unemployment and financial hardship as her source of poor mental health, despite co-habiting with her perpetrator.

“We live off of Centrelink payments. Life here is expensive and it’s hard to keep up on just government allowance”

– Survivor from Syria

The focus on structural forms of violence alludes to the significant impact that structural violence has on the day-to-day lives of newly arrived refugee women regardless of their marital status. Service providers echoed this sentiment, regularly describing that their clients often prioritise support for structural factors (such as risk of homelessness, precarious visa status, and poverty) over accessing support for the IPV they are currently or have previously experienced.

“For some clients [forced migrant women], the challenge of resettlement in itself is big enough. Then, to face another challenge of doing it on your own or having to go through a legal battle, it’s extremely stressful. Some clients opt to just stay where they are because the alternative would be too much”

– Service Provider

This section illustrates the persistence of structural and symbolic violence against forced migrant women and thus the importance of addressing structural and symbolic forms of violence against forced migrant women regardless of whether they are currently in an IPV situation or not.

### The importance of autonomy and independence

Most women expressed that one of the largest impacts of violence on their mental health stemmed from feelings of being stripped of their autonomy. For example, in the quote below, a workshop participant describes how a lack of grasp of the English language and the lack of appropriate interpretation support (a form of structural violence) made her feel like she was stripped of her autonomy and independence.

“Because I don’t speak English, I can’t defend myself. When I first came to Australia, I was physically abused by my husband. When the police came, he told them that I hit him. I had no idea what they were saying so I just kept saying ‘yes, yes, yes’ so they thought that I was the one that had hit him. When the police eventually found out the truth, they said ‘how could this happen to you?’ and I replied ‘Because I don’t speak English’”

– Workshop Participant

This same woman was later interviewed, and during the interview, she expressed a feeling of frustration at not being heard due to lack of appropriate interpretation services (a form of structural violence).

“If you want to be heard, you have to speak English and interpreters often get it wrong. I understand what they’re saying but I can’t say anything because my English is poor, but I can tell that they’re getting it wrong”

– Survivor from Lebanon

Conversely, many survived described gaining independence as having a positive impact on their mental health. In the quotes below, interview participants speak about improvements in their mental health upon ganing independence.

“My wellbeing is better since I left him, and the financial independence I now have, plays a big part in this”

– Survivor from Lebanon

“The more independence I got, the better I felt”

– Survivor from Lebanon

The evidence provided in this section illustrates the importance of autonomy and independence on the mental health of forced migrant women: when women are stripped of control over their lives, it negatively impacts their mental health and, conversely; when women gain independence, their mental health and wellbeing improves.

## Discussion

This article is among the first to document the intersections of different forms of violence as they relate to the lived experiences of forced migrant women. More specifically, we show how structural and symbolic forms of violence during resettlement increase the risk of IPV for forced migrant women. Furthermore, we show that even when forced migrant survivors leave IPV situations, structural and symbolic violence persist and exacerbate the mental ill-health risks and consequences of IPV. These findings build on and expand on recent literature that considers multiple contexts of forced migration (not only resettlement) and provides a broad overview of the compounding and intersecting forms of gendered violence against forced migrant women [14, 18]. More specifically, this article highlights the important role that autonomy and independence play in the mental health and wellbeing of forced migrant women. This ties into broader concepts of structural and symbolic violence and IPV by revealing how different forms of violence exert control through power imbalances that necessarily strip victims of their autonomy. For example, unemployment due to visa status is a specific form of unemployment that impacts forced migrants and leads to over-reliance on the state and NGOs for financial support, thus stripping forced migrants of economic independence. Similarly, experiences of IPV are often driven and underpinned by a perpetrator’s need for control which necessarily requires victims to lose physical and/or financial and/or social control over their lives. Finally, symbolic violence creates differential access to power (e.g., through racism or patriarchy) which determines who controls and who is controlled.

### Implications for policy and practice

At a practice level, our findings suggest that IPV programs for forced migrant women must address structural and symbolic violence as part of their programming. Given the persistent nature of structural and symbolic violence, settlement, migration, violence, and mental health services must also provide support and programmes to address structural and symbolic violence outside the limitations of IPV. For example, mental health programs with survivors of IPV need to also address the mental health impacts of housing, unemployment, and precarious visa status. Similarly, violence services must also work to address symbolic factors with examples including (but not limited to) programs addressing patriarchy with newly arrived communities and racism within host communities.

At a policy level, services and organisations must also use their power to advocate and lobby for cross-sector and cross-governmental policy change. Ultimately, structural violence is perpetrated by state institutions which reinforce and reproduce symbolic power relations. With this in mind, it is the responsibility of state actors to put policies in place that not only eliminate structural harm that is perpetrated against forced migrant women, but also works to mend the existing harm that has been perpetrated.

### Limitations of the study

The evidence presented in this article is limited to experiences of 24 forced migrant women and 4 mental health service providers in Melbourne, Australia. Caution should thus be made to applying these findings to other places in Australia or other cities of resettlement. This research would be further strengthened and complimented by other studies that adopt a similar approach in other resettlement contexts around the world.

The focus on forced migrant women also means that this research necessarily conceptualised gender in binary terms and did not explore the experiences of male or lesbian, gay, transgender, queer (LGBTQ) forced migrants. This research would thus be further strengthened by research investigating the experiences of male and LGBTQI forced migrants as this would provide a more robust understanding of how patriarchy and racism interact in the context of forced migration. Understanding such complexities would enable more effective recommendations to be made about how policy, programs and practices can be designed and delivered to challenge oppressive structures.

## Conclusions

Our findings reveal that there is a complex and inter-dependent relationship between structural, symbolic, and intimate-partner violence against forced migrant women during resettlement, all of which compound the mental ill-health causes and consequences. Our findings subsequently add to the existing body of evidence on the intersecting nature of different forms of violence against forced migrant women and extend this to the mental health implications for forced migrant survivors of IPV during the resettlement period. To address the structural and symbolic drivers of mental ill-health for forced migrant women who have experienced IPV, there must first be recognition from service providers and policymakers of how the resettlement context exacerbates mental ill-health causes and consequences for forced migrant women who have experienced IPV. There must then be more collaborative efforts among and between government and service providers to address structural, symbolic, and intimate-partner violence against forced migrant women. Further research must also be undertaken to include the experiences of male and LGBTQ forced migrants to gain a more holistic picture of how racism and patriarchy interact in the resettlement context.

## Supplementary Information

**Additional file 1.** Service Provider Interview Guide.

**Additional file 2.** English Translations of Workshop Materials.

**Additional file 3.** Survivor Interview Guide.

**Additional file 4.** Coding Framework.

## Data Availability

The datasets presents in this manuscript are not readily available because of the sensitive nature of the data meaning there are ethical requirements that stipulate data cannot be shared.

## Abbreviations

HSP: Humanitarian settlement program
IPV: Intimate partner violence
LGBTQ: Lesbian, gay, bisexual transgender
