# Supplementary material for: How structural and symbolic violence during resettlement impacts the social and mental wellbeing of forced migrant women: the lived experiences of Arabic speaking survivors of IPV resettled in Melbourne, Australia
Source: Confl Health. 2022 Nov 11;16:59. doi: 10.1186/s13031-022-00494-6 (PMC9652810; doi:10.1186/s13031-022-00494-6)
Supplement: Supplementary file 1 — Additional file 1. Service Provider Interview Guide. [file 13031_2022_494_MOESM1_ESM.pdf]

Additional File 1: Interview guide for interviews with mental health service providers

Theme	Prompts / example questions
Services offered	Can you tell me a little bit about yourself and your role here?<br>What type of supports does your service offer to refugee communities?<br>Are there specific supports that your service offers to refugee women?<br>What do refugee women using your service come for help with?
Perspectives and experience in relation to violence against women	Can you tell me a bit about what impact you think of experiences of torture or trauma might have on mental health? How does this affect relationships within the family?<br>Is violence against women – whether that be family violence, or other forms of violence against women – something that you or your colleagues suspect very often (even if you do not receive direct disclosures)?<br>In the past, if you suspected that a client was experiencing violence, but this had not been disclosed to you, what did you do?<br>Do you yourself have much contact with refugee women experiencing violence? Or are other colleagues within the organisation more likely to have direct contact with women experiencing violence?
Pathways to support	How much do you think your clients know about support services for violence?<br>Do you think your clients access violence-specific services very often or use other sources of support?<br>Have you had any experience in referring clients to violence-specific support services?
Barriers and facilitators to providing support and to preventing violence against women	Your organisation plays a key role in supporting refugees who have experienced some times quite extreme violence in the past. What do you think the role of your organisation is in relation to women who may be experiencing violence now?<br>What do you think makes it easier for you or your organisation to provide support to refugee women experiencing violence, or to contribute to prevention of violence against women?<br>What do you think makes it more challenging for you or your organisation to provide support to refugee women experiencing violence, or to contribute to prevention of violence against women?
Professional development support	Have you received any training in relation to family violence or violence against women?<br>Is there organisational support for you if you are trying to assist women experiencing violence? What does this look like?
Suggestions	Is there anything you would like to add to help us better understand your views on the role of specialist refugee mental health services in supporting women experiencing violence and/or preventing violence against refugee women?
