# Supplementary material for: How structural and symbolic violence during resettlement impacts the social and mental wellbeing of forced migrant women: the lived experiences of Arabic speaking survivors of IPV resettled in Melbourne, Australia
Source: Confl Health. 2022 Nov 11;16:59. doi: 10.1186/s13031-022-00494-6 (PMC9652810; doi:10.1186/s13031-022-00494-6)
Supplement: Supplementary file 2 — Additional file 2. English Translations of Workshop Materials. [file 13031_2022_494_MOESM2_ESM.pdf]

Additional File 2: Participatory Workshop Activities (translated to English)

Activity 1: Life in Australia

Good Things	Challenging things

Men		Women	
Good things	Challenging things	Good things	Challenging things

Activity 2: Mental Health

What makes you happy here?	What makes you sad here?

What are the idioms or expressions you use to express those feelings and to whom?	
Idioms or expressions	To whom?

Activity 3: Healthy Families

The tree root represents the causes, the tree trunk represents the family and the tree leaves represent the consequences / effects.

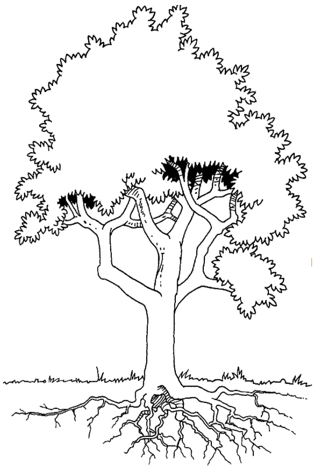

Happy, healthy family

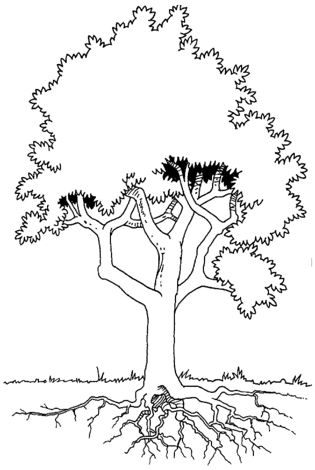

Unhappy, unhealthy family
