# Supplementary material for: How structural and symbolic violence during resettlement impacts the social and mental wellbeing of forced migrant women: the lived experiences of Arabic speaking survivors of IPV resettled in Melbourne, Australia
Source: Confl Health. 2022 Nov 11;16:59. doi: 10.1186/s13031-022-00494-6 (PMC9652810; doi:10.1186/s13031-022-00494-6)
Supplement: Supplementary file 3 — Additional file 3. Survivor Interview Guide. [file 13031_2022_494_MOESM3_ESM.pdf]

Additional File 3: Interview guide for interviews with survivors (translated to English)

Theme	Prompts / example questions
Process of migration and mobility	How did you end up coming to Australia?<br>Where did you first arrive in Australia and when?<br>When did you come to the place you are living at now?
Life in Australia	What is it like for you living here now?<br>What are the good things?<br>What are the difficult things?<br>Are there many people from [country of origin] living near here?<br>Do you have friends or support from the community?<br>Do work or go to school?
Family / community structure	Could you describe your family structure?<br>Are you with your partner?<br>How long have you been with your partner?<br>How did you meet your partner?<br>Do you have children?<br>Are there extended family members in Australia too? Do they live with you?
Impact of immigration on family	How has coming to Australia affected your family/relationship?<br>Are their positive impacts?<br>Have your relationships or responsibilities changed? Are there any new challenges, stress or difficulties?<br>Has what is expected of women and men in your family and community changed since you settled in Australia?<br>How do the women and men in your family and community feel about these changes?
Mental health and wellbeing, mental ill-health and distress	What do you think makes an individual and family happy and healthy?<br>The refugee journey is not an easy one, and many people can feel distressed, sad or worried for a long time afterwards. Is this something that you talk about in your family or community?<br>How has this affected your family?
Family violence	What do you think makes a family relationship healthy and respectful?<br>Women across the world can experience violence in their home or from members of their family. Is this something that is talked about in your community?<br>Has this affected you or your family?
Interaction between mental ill-health, family violence and help-seeking	Family violence is something that affects people from all walks of life, in all countries of the world. But there are things that can make it worse or better. Some people who work with refugee families think that there might be a relationship between the distress, sadness and worry people experience as a refugee and family violence. What do you think?<br>Does being distressed or worried or angry all the time have an effect on family violence?<br>How does being distressed or worried influence whether and where people seek help?
Personal experiences of services	When you needed help, where did you go?<br>Can you tell me a bit more about what that was like?

	What things make it easier to get help? Are there things that make it difficult?<br>Did your husband ever seek help? Where did he go?
Strengths and resilience	It takes a strong person to cope with the difficult things you have talked about. What do you think helps you to cope? Are there things that could be done by government or services that would make it easier for you to cope?
