# Supplementary material for: How structural and symbolic violence during resettlement impacts the social and mental wellbeing of forced migrant women: the lived experiences of Arabic speaking survivors of IPV resettled in Melbourne, Australia
Source: Confl Health. 2022 Nov 11;16:59. doi: 10.1186/s13031-022-00494-6 (PMC9652810; doi:10.1186/s13031-022-00494-6)
Supplement: Supplementary file 4 — Additional file 4. Coding Framework. [file 13031_2022_494_MOESM4_ESM.docx]

## Additional File 4: Coding framework for thematic analysis of data

| **Organising theme** | **Basic theme** | **Sub-theme(s)** | | | |
| --- | --- | --- | --- | --- | --- |
| 1. Life in Australia | A. Good things about life in Australia | The importance of being in a safe place | | | |
|  |  | Availability of support and services | | | |
|  |  | Living in a rights-respecting environment | | | |
|  | B. Challenges of life in Australia | Experiences of racism in public places | | | |
|  |  | Trouble securing employment | Due to family commitments | | |
|  |  |  | Due to inability to convert qualifications | | |
|  |  |  | Due to low proficiency in English | | |
|  |  | Lack of community support | Due to distance from family | | |
|  |  |  | Due to lack of existing community | | |
|  |  | Navigating new systems or services | | | |
|  |  | Reliance on English language | | | |
| 2. Family, community and marriage norms | A. Marriage | Getting married | Family pressure to get married (especially from male members) | | |
|  |  |  | Forced and early marriage | | |
|  |  |  | Being reprimanded for refusing family marriage advice | | |
|  |  | Problems with the marriage | Abuse from extended family (especially female members) | | Physical abuse |
|  |  |  |  |  | Emotional abuse |
|  |  |  | Husbands being unfaithful | | |
|  |  | Pressure to stay in marriage | Family pressure to stay in marriage | | |
|  |  |  | Cultural pressure to stay in marriage due to stigma | | |
|  |  |  | Religious pressure to stay in marriage e.g. refusal of divorce by religious leaders | | |
|  |  |  | Barriers to leaving the relationship | | |
|  | B. Determinants of a happy family | Limited extended family intervention | | | |
|  |  | Appropriate marriage match | | | |
|  |  | Stability | Housing stability | | |
|  |  |  | Family reunification | | |
|  |  | Importance of family unit | | | |
|  | C. Cultural norms | Gender norms | | | |
|  |  | Age norms | | | |
|  | D. Normalisation of violence | | | | |
| 3. Impact of immigration | A. Impact on relationship | Boredom and lack of work resulted in tension | | | |
|  |  | Unfaithfulness beginning after immigration | | | |
|  |  | Changes in violence | | | |
|  | B. Decreased independence | Reliance on others | Reliance on others to communicate due to poor English | | |
|  |  |  | Relying on others to get around due to inability to drive | | |
|  |  | Precarious visa status | | | |
|  |  | Inability to work | | | |
|  | C. Negative impact on mental health | | | | |
|  | D. Impact on children | Child custody changes upon arrival | | | |
|  |  | Impacts on education | Inconsistent schooling | | |
|  |  |  | Lack of understanding of the school system here | | |
| 4. Mental health and wellbeing | A. Victim’s mental health | Impact of relationship on mental health | | | |
|  |  | Impact of structural violence | Settlement stressors | Unemployment and financial instability | |
|  |  |  |  | Housing | |
|  |  |  |  | Loneliness or thinking of family back home | |
|  |  |  | Stressors along the refugee journey | | |
|  |  | Importance of independence for mental health | | | |
|  |  | Coping strategies | Developed on their own | | |
|  |  |  | Developed with a psychologist | | |
|  |  | Access to Mental Health services | | | |
|  |  | Lack of access to Mental Health services | Due to lack of awareness of available services | | |
|  |  |  | Due to stigma around mental ill-health and accessing services | | |
|  |  |  | Due to not seeing the value of mental health services | | |
|  | B. Perpetrator’s mental health | Anger / mood swings | | | |
|  |  | Poor mental health due to loss of control e.g. inability to support family | | | |
|  |  | Substance abuse (alcoholism and gambling) | Physical abuse when drunk | | |
|  |  |  | Financial abuse to fund addiction | | |
|  |  | Inconsistent access to services | | | |
|  | C. Ongoing impact of violence on children’s mental health | | | | |
|  | D. Mental Health service delivery | Services provided | | | |
|  |  | Entry point | | | |
|  |  | Disclosure of Family Violence | | | |
|  |  | Barriers to access | | | |
|  |  | Limitations & improvement opportunities | | | |
|  | E. Impact of trauma or mental ill-health on family unit | | | | |
| 5. Family violence | A. Control | Control of communication | Not allowing wife to take English lessons | | |
|  |  |  | Refusing to translate or interpret e.g. mail from Centrelink in English | | |
|  |  |  | Speaking in English in front of wife so that they would not understand | | |
|  |  | Control of movement | Not allowing wife to take driving lessons | | |
|  |  |  | Not allowing wife to leave the house | | |
|  |  |  | Not allowing wife to see her family | | |
|  |  |  | Stalking | | |
|  | B. Abuse | Financial abuse | Withholding money | | |
|  |  |  | Withholding information on household finances | | |
|  |  |  | Punishment by not paying rent or bills | | |
|  |  |  | Not financially supporting children | | |
|  |  |  | Stealing money from wife e.g. to buy alcohol | | |
|  |  | Physical abuse | Hitting | | |
|  |  |  | Stalking | | |
|  |  |  | Throwing things | | |
|  |  |  | Occurrence | | Started early in marriage |
|  |  |  |  |  | Got worse during pregnancy |
|  |  | Emotional abuse | Bullying | | |
|  |  |  | Lying | | |
|  |  |  | Shouting | | |
|  |  |  | Threats | | |
|  |  |  | Disrespect | | |
|  |  |  | Embarrassment in front of others | | |
|  |  | Sexual abuse | | | |
|  | C. Impact on children | Children witnessing violence | | | |
|  |  | Children playing an active role in trying to stop the violence e.g. by calling the police | | | |
|  | D. Urgency of leaving the relationship | | | | |
| 6. Support and services | A. Community support | Positive experiences | Awareness of services from community members | | |
|  |  |  | Assistance from members of the community | | Financial assistance |
|  |  |  |  |  | Assistance on how to use services |
|  |  |  | Preference for community support | | Due to better understanding of culture and norms |
|  |  |  |  |  | Due to distrust of services |
|  |  | Negative experiences | Lack of community support because of community members’ own individual struggles | | |
|  |  |  | Unwanted community interference in their lives | | |
|  | B. Government and non-profit services | Social services | Accessibility | | Access to a range of services |
|  |  |  |  |  | Lack of awareness about what services are available |
|  |  |  |  |  | Lack of services during transition periods |
|  |  |  |  |  | Existence of so many services can be overwhelming |
|  |  |  |  |  | Misconceptions about social services |
|  |  |  | Examples of a single social worker, interpreter, etc. going above and beyond | | |
|  |  |  | Importance of cultural competence | | |
|  |  | Employment and financial assistance services | Put a lot of pressure on you to find work | | |
|  |  |  | Force you to work for free to gain experience that you already have | | |
|  |  |  | Reliance on financial assistance | | |
|  |  | Language services | English lessons | | Importance of language lessons for independence |
|  |  |  |  |  | Competing priorities |
|  |  |  |  |  | Not enough government funded English lessons |
|  |  |  | Interpreter services | | Positive experiences with interpreters |
|  |  |  |  |  | Negative experiences with interpreters |
|  |  | Housing services | High cost of rent | | |
|  |  |  | Moving house regularly | | |
|  |  |  | Government housing not being prioritised correctly | | |
|  |  | Law enforcement services | Police filing intervention orders | | |
|  |  |  | Discrimination faced by law enforcement | | |
|  |  |  | Justice system difficult to navigate | | |
|  |  |  | Language barriers when communicating with the police, courts, etc. | | |
